# My mind is still mine: a self-portrait in a photography project for adolescents and young adults with cancer

**DOI:** 10.1186/s12904-021-00789-0

**Published:** 2021-07-14

**Authors:** Andrea Ferrari, Alice Patriccioli, Matteo Silva, Matteo Davide Bonvicini, Maura Massimino

**Affiliations:** Pediatric Oncology Unit, Fondazione IRCCS Istituto Nazionale dei Tumori, Via G. Venezian, 1 -20133 Milan, Italy

**Keywords:** Adolescents, Cancer, Youth Project, Photography, Art, Psychological support

## Abstract

This commentary describes the unusual self-portrait contributed by a 26-year-old receiving treatment for relapsing medulloblastoma to a photography project undertaken by a group of patients as part of the Youth Project, a scheme dedicated to young cancer patients with the dual aim of optimizing medical aspects of their care and promoting a holistic approach to their needs. The article briefly describes how creative projects can play an important part in giving young people with cancer new ways to tell their stories and express their feelings. There is still a limited understanding of the specific needs of adolescents and young adults with cancer, and it is important to draw attention to them and to the need to devise a person-centered approach to cancer patients in this age group.

## Background

A cancer diagnosis is traumatic and difficult to face at any age, but may be particularly challenging when it happens to young people. It can disrupt several delicate developmental processes underway in adolescence – a time for establishing identities, developing social relationships, gaining independence, or experimenting sexually, just to give a few examples. The psychosocial sphere of adolescents and young adults with cancer may be particularly fragile, giving rise to emotional, psychological and social challenges that healthcare providers need to bear in mind [1, 2].

## Main text

“*This is my self-portrait for today*, 

*It shows the very essence of me.*

*The title of my photo can be*

*‘I will try to fix you’*,

*like that song by Coldplay.*

*People are defined by their thoughts*,

*by their minds.*

*Their body is just a shell.*

*For almost any part of your body, you can get a replacement*,

*be it organic or artificial*,

*a transfusion, a transplant, a prosthesis.*

*But you’re still you*,

*just with something added - or taken away*,

*depending on how you look at it.*

*But you can’t replace your brain.*

*In my mind, lots of people have come*

*to “try to fix me” in my time of need*,

*to help me feel better.*

*But my brain has stayed the same.*

*My mind is still mine*.”

**Fig. 1:**
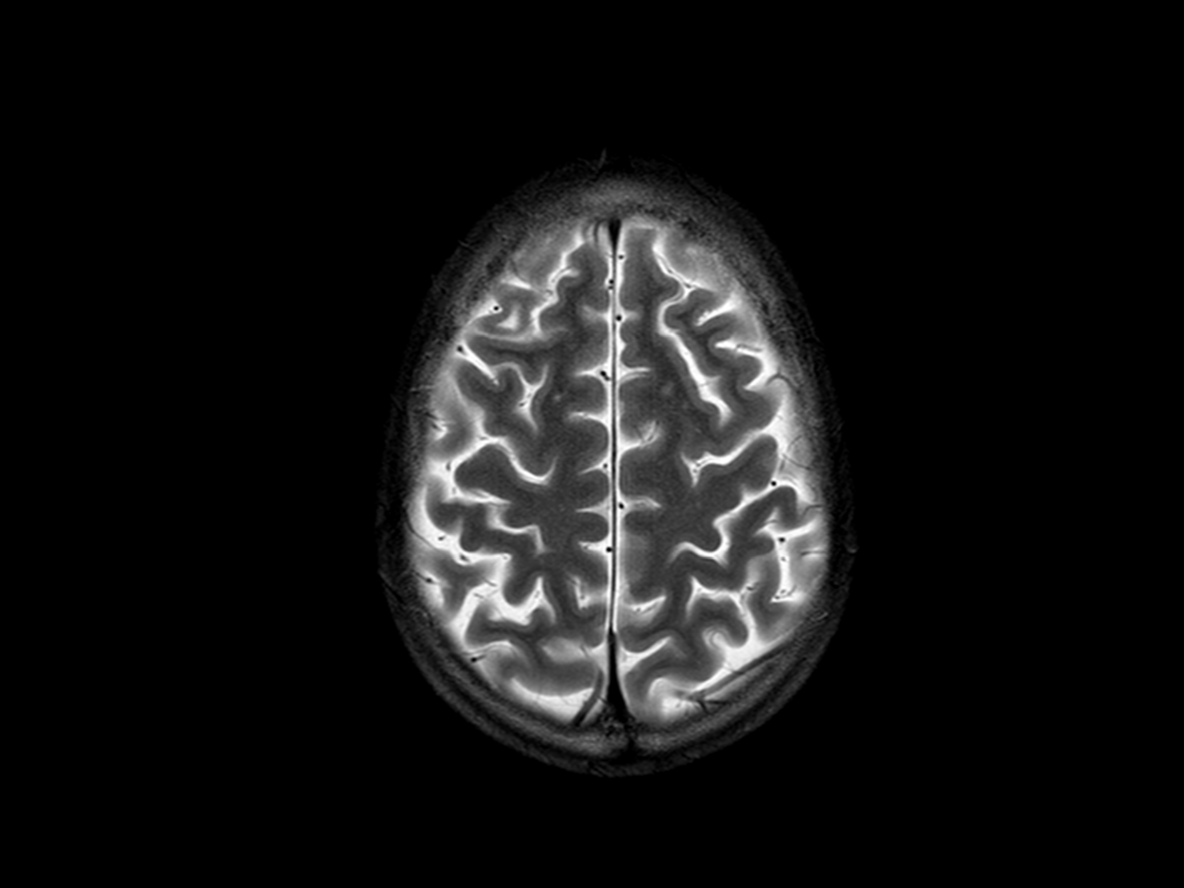
The photo B. submitted of his brain MRI scan as a self-portrait for a photography project entitled “*Looking out to see within*”

These are the words B. used to accompany a photo - an MRI (magnetic resonance imaging) scan of his brain – that he presented as a self-portrait for a photography project (Fig. 1).

B. is now 26 years old. He is currently receiving treatment at our institute’s pediatric oncology unit for a third relapse of his medulloblastoma, which was first diagnosed at 13 years of age. Over the years, he has been involved in many activities organized by the Youth Project, a scheme dedicated to young cancer patients with the dual purpose of optimizing medical aspects of their care while also paying attention to their quality of life (www.ilprogettogiovani.org) [3]. Launched in 2011, the Youth Project provides age-appropriate spaces in hospital and organizes creative activities that give patients special ways to express themselves more freely, to tell us about their fears and their hopes. Over the years, various projects involving photography, music, creative writing, and fashion design have been developed and reported [4–9]. The last creative laboratory organized as part of the Youth Project was a photography project entitled “*Looking out to see within*”, run by a professional photographer from May to September 2020 [10]. B. took part in this project together with another 24 patients. As in previous projects, the patients who participated were adolescents and young adults (in principle aged 15–25 years, but the age limits were not strict) receiving treatment at the Pediatric Oncology Unit of the Istituto Nazionale dei Tumori in Milan, or those who had completed their treatments. No further specific inclusion/exclusion criteria were applied. Patients were invited to join this initiative by the Youth Project team (which includes dedicated doctors, psychologists and a youth worker) and were entirely free to do so or not. Patients (or their parents or legal guardians for those underage) gave their written informed consent to their involvement in the project. Participants met weekly on a digital platform to comply with social distancing rules due to the COVID-19 pandemic. The self-portrait was one of several themes covered by the photography project (the others were: the window through which we can look out on the world; and places and people we meet when we leave our room to go outside). As self-portraits, most participants submitted selfies, but B. sent a picture of his MRI. He said, “*I can’t imagine a better way to see inside myself*”.

His words and his photograph exemplify many of the traits that we have seen in our adolescent and young adult patients working on these creative projects over the years. Our young participants in the Youth Project often reveal a sense of humor (a brain MRI as “*my self-portrait for today”)*, a poetic spirit, an urge to speak freely about themselves, and a strong sense of self-determination (“*my mind is still mine*”). We believe that these characteristics, together with a self-deprecating attitude that we have often noted, may be fundamentally important to patients having to cope with the trauma of a diagnosis of cancer and its treatment at such a delicate developmental age.

## Conclusions

We have learned from B. and other patients like him how important it is to organize projects and activities for our young patients to help them address the psycho-social challenges posed by their disease, and give them a chance to freely “voice” their fears and hopes. Far from being considered as “art therapy” (which relies on specific theoretical models), such creative projects should be seen as a form of psychological support, complementary to other more traditional approaches. They can open precious windows onto the inner worlds of our patients, enabling us to gain a better understanding of what they are really thinking and feeling, directly from their own words and artistic output. We have learned that creative projects have the potential to provide a much more genuine picture than we might obtain from structured specialist interviews. We believe that giving our patients the opportunity to freely “voice” their feelings is a fundamental part of helping them to process what is happening to them, to accept it and find the resources they need to cope with the whole period of their treatment in as positive a manner as possible. Using their creative spirit and art as a vehicle can make it easier for them to give vent to strong emotions, enabling them to express themselves in a more natural, less “controlled” way.

## Data Availability

Not applicable.
